# SHH medulloblastoma and very early onset of bowel polyps in a child with *PTEN* hamartoma tumor syndrome

**DOI:** 10.3389/fnmol.2023.1228389

**Published:** 2023-08-24

**Authors:** Anna Maria Caroleo, Silvia Rotulo, Emanuele Agolini, Marina Macchiaiolo, Luigi Boccuto, Manila Antonelli, Giovanna Stefania Colafati, Antonella Cacchione, Giacomina Megaro, Andrea Carai, Maria Antonietta De Ioris, Mariachiara Lodi, Assunta Tornesello, Valeria Simone, Filippo Torroni, Giuseppe Cinalli, Angela Mastronuzzi

**Affiliations:** ^1^Department of Onco-Hematology, Cell Therapy, Gene Therapy and Hemopoietic Transplant, Bambino Gesù Children’s Hospital (IRCCS), Rome, Italy; ^2^Department of Pediatrics, Sapienza University of Rome, Rome, Italy; ^3^Laboratory of Medical Genetics, Translational Cytogenomics Research Unit, Bambino Gesù Children’s Hospital, IRCCS, Rome, Italy; ^4^Rare Diseases and Medical Genetics Unit, IRCCS Bambino Gesù Children’s Hospital, Rome, Italy; ^5^School of Nursing, College of Behavioral, Social and Health Sciences Healthcare Genetics Interdisciplinary Doctoral Program, Clemson University, Clemson, SC, United States; ^6^Faculty of Medicine and Dentistry, Department of Radiological, Oncological, and Pathological Anatomy Sciences, Sapienza University of Rome, Rome, Italy; ^7^Neuroradiology Unit, Department of Imaging, Bambino Gesù Children’s Hospital (IRCCS), Rome, Italy; ^8^Neurosurgery Unit, Department of Neurosciences, Bambino Gesù Children’s Hospital (IRCCS), Rome, Italy; ^9^Pediatric Oncology Unit, Ospedale Vito Fazzi, Lecce, Italy; ^10^Digestive Endoscopy and Surgery Unit, Bambino Gesù Children Hospital, IRCCS, Rome, Italy; ^11^Pediatric Neurosurgery Unit, Department of Neuroscience, Santobono-Pausilipon Children’s Hospital, Naples, Italy

**Keywords:** cancer predisposition syndrome (CPS), pediatric, PTHS, medulloblastoma (MB), intestinal polyp, *PTEN* hamartoma tumor syndrome

## Abstract

Phosphatase and tensin homolog (*PTEN*) hamartoma tumor syndrome (PHTS) is a cancer predisposition syndrome characterized by an increased risk of developing benign and malignant tumors, caused by germline pathogenic variants of the *PTEN* tumour suppressor gene. *PTEN* gene variants often present in childhood with macrocephaly, developmental delay, and/or autism spectrum disorder while tumors and intestinal polyps are commonly detected in adults. PHTS is rarely associated with childhood brain tumors with only two reported cases of medulloblastoma (MB). We report the exceptional case of an infant carrying a germline and somatic pathogenic variant of *PTEN* and a germline and somatic pathogenic variant of *CHEK2* who developed a MB SHH in addition to intestinal polyposis.

## 1. Introduction

Phosphatase and tensin homolog (*PTEN*) hamartoma tumor syndrome (PHTS) is a rare neurocutaneous syndrome caused by germline pathogenic variants of the *PTEN* tumor suppressor gene (Hendricks et al., 2020; Isik et al., 2020; Kim et al., 2020) that cause an increased risk of developing benign and malignant tumors of the thyroid, breast, endometrium, skin, and brain. In addition to cancer susceptibility, PHTS features include macrocephaly, autism spectrum disorder, atypical neurodevelopment, benign thyroid lesions, and dermatologic findings (trichilemmomas, papillomas). PHTS may be considered a non-classical brain tumor polyposis syndrome, as central nervous system (CNS) manifestations are a rare component of the patient’s clinical burden (Kim et al., 2020). It is a rare disease with an estimated prevalence of 1/200.000, but it is probably underestimated because most patients are not recognized as such (Nelen et al., 1999; Ngeow and Eng, 2015; Karczewski et al., 2020). Approximately 50% of PHTS cases are inherited in an autosomal dominant manner, with the remainder of cases having a *de novo* mutation; in approximately 80% of case mutations of the *PTEN* gene affects the germline (Kim et al., 2020). All types of pathogenic variants (loss-of-function, deletions, missense, and promoter abnormalities) have been reported with no clear genotype–phenotype correlation (Smith et al., 2019). *PTEN* gene mutations show age-related penetrance (Lachlan et al., 2007): in childhood, they are often associated with macrocephaly, developmental delay (DD), and/or autism spectrum disorder, less commonly with thyroid lesions, while the development of tumors and intestinal polyps are rare, being more frequently detected in adult individuals (Heald et al., 2010; Hansen-Kiss et al., 2017; Ciaccio et al., 2019; Macken et al., 2019).

Medulloblastoma (MB) is a heterogeneous tumor that represents about 10% of CNS malignancies in children between 0 and 14 years of age (Millard and De Braganca, 2016). There are four MB subgroups (Sonic Hedgehog or SHH, WNT, group 3, and group 4), which are associated with specific transcriptional, epigenetic, and clinical characteristics (Vladoiu et al., 2019). However, the molecular details of each subgroup are not fully understood to date (14). Recently, it has been shown that the SHH subgroup is most frequently (approximately 20–40%) associated with germline mutations (*BRCA2*, *PALB2*, *PTCH1*, *SUFU*, and *TP53*; Waszak et al., 2018; Garcia-Lopez et al., 2021). Currently, MB cases are rarely described in individuals with Cowden syndrome (Waszak et al., 2018; Tolonen et al., 2020), a condition included in the PHTS spectrum. Instead, cases associated with other CNS tumors such as dysplastic gangliocytoma, meningioma, pineal tumor, oligodendroglioma, and glioblastoma have been reported in patients with this condition (Kim et al., 2020).

Susceptibility to develop intestinal polyps is one of the most distinctive features of PHTS, involving up to 95% of PHTS patients throughout life (Heald et al., 2010). Bowel polyps may be found from the stomach to the colon, and histology may include hamartomatous polyps (most common), ganglioneuromas, adenomas, and inflammatory polyps (Heald et al., 2010). This clinical manifestation is similar to Juvenile Polyposis Syndrome (JPS): hamartomatous polyps are indistinguishable (Schreibman et al., 2005) but tend to occur in adulthood (Lachlan et al., 2007; Heald et al., 2010). While the increased risk for the development of breast and thyroid cancers is well documented, the development of hamartomatous polyps does not lead to an increased risk of colorectal cancer. Heald et al. documented that colorectal cancer occurred in 7.1% of cases of their series (Heald et al., 2010).

Here we report the first pediatric case of PHTS with both a germline and somatic variant in *PTEN* and in *CHEK2*, who presented a significantly early onset of MB SHH (15 months), in addition to a remarkably early picture of hamartomatous intestinal polyposis.

## 2. Materials and methods

The patient and his legal guardians conferred informed consent for the study. A centralized review of histological characterization was performed. Molecular genetics studies were performed on genomic DNA extracted from peripheral blood using a next-generation sequencing (NGS) panel including medulloblastoma and cancer predisposition genes (*APC*, *BRCA2*, *PALB2*, *PTCH1*, *PTCH2*, *SUFU*, *PTEN*, *TP53*, *CHEK2*, and *GPR161*), according to the manufacturer’s protocol (Twist Bioscience, CA, USA). The presence of deletions and duplications in *PTCH1* and *SUFU* genes on peripheral blood was also excluded by multiplex ligation-dependent probe amplification (MLPA) according to the manufacturer’s protocol (MRC Holland, Amsterdam, Netherlands).

## 3. Case report

The patient is a Caucasian male, referred to the Bambino Gesù Children’s Hospital at 15 months of age after the removal of a cerebellar mass, histologically compatible with MB at another center. He is the firstborn child to unrelated parents. His family history is free of neurocognitive developmental alterations, his father has intestinal polyposis, his paternal grandfather and uncle died of intestinal cancer; his paternal grandmother died of pancreatic cancer. He was born at 39 weeks of gestational age after an uneventful pregnancy. His birth weight (3,700 gr, 60 percentile, +0.79 SD) and height (50 cm, 20 percentile, −0.9 SD) were normal, while his head circumference was above normal (38 cm, 98 percentile, +3.0 SD). On arrival to the hospital, at the age of 15 months, he presented with macrocephaly (+3.0 SD) and psychomotor delay with major weaknesses related to language skills as detected by Griffiths Developmental Scales.

**Figure 1:**
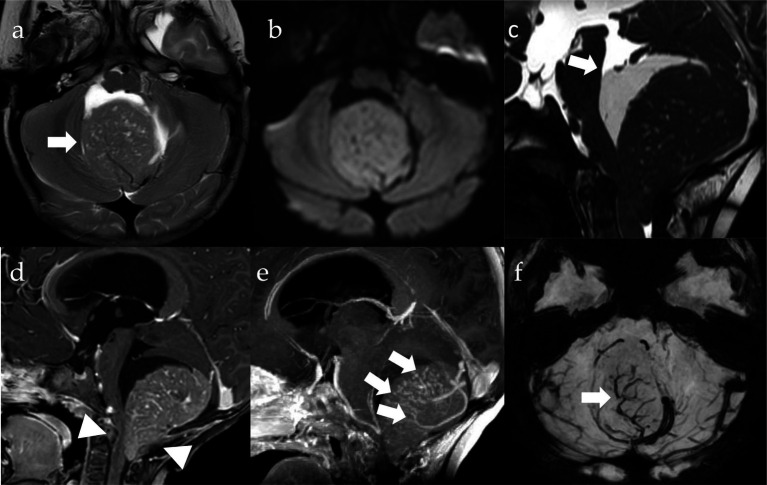
MRI imaging. Pre-operative axial T2w **(A)**, DWI (diffusion weighted imaging, **B**) and SWI (susceptibility weighted imaging, **F**) images, sagittal CISS (three-dimensional constructive interference in steady state, **C**) Gd T1w **(D)** and MIP (maximum intensity projection, **E**) images. There is a well-circumscribed lesion in the posterior fossa (**A**, arrow), which is centered on the cisterna magna and pushing the vermis cranially, with growth into the fourth ventricle and extension through the foramen of Magendie onto the posterior aspect of the upper cervical cord (**D**, arrowheads). The tumor is isointense to the cerebellar cortex on T2 **(A)** and shows restriction of diffusivity **(B)** due to high cell density along with high nuclear-to-cytoplasmic ratio **(B)**. There is significant contrast-enhancement **(D)** and intralesional vessels (**E,F**, arrows). Cystic components are appreciable and appear larger in the cranial portions of the lesion (arrow, **D**).

The surgical removal was fraught with difficulty, despite neuroimaging suggested a superficial, almost extra-axial lesion. The tumor was in fact very hard and bled profusely, to the point of reminding more of a hemangioblastoma, with a complex pattern of intratumoral vessels, than of an MB, which was moreover completely isodense at the pre-operative computed tomography (CT) scan. Complete resection was confirmed by postoperative magnetic resonance imaging (MRI) (Figure 1). Cerebrospinal fluid was free of neoplastic cells.

The histological examination revealed an embryonic neoplasm characterized by the presence of nodular and internodular areas. The nodular areas showed elongated aspects and consisted of neurocytic-type cells immersed in a fibrillar stroma. In the internodular areas, the cells were markedly hyperchromic with frequent mitosis. The immunohistochemical investigation showed a pattern coherent with MB SHH. The cells were positive for synaptophysin in the nodular areas; positivity was observed for GAB1, YAP1, and Filamin A. The proliferation index evaluated with Ki67 was high in the internodular areas (about 30%).

Molecular genetic characterization by NGS was performed on genomic DNA extracted from circulating leukocytes of the patient and unaffected parents to check for the presence of germline variants in high-risk cancer-predisposition genes. None of the genes typically associated with MB (*APC*, *BRCA2*, *PALB2*, *PTCH1*, *SUFU*, and *TP53*) (Waszak et al., 2018) were found to be mutated. Sequence analysis showed a germline heterozygous variant c.79 T > A in the *PTEN* gene (NM_000314.6) determining the missense change p.Tyr27Asn (rs746128825), previously reported in association with PHTS (Ngeow et al., 2014). This single-base substitution affects the last nucleotide position of the exon 1 and could be a splicing variant. However, further RNA studies are needed to test this hypothesis but are not feasible at present due to sample unavailability. This variant can be classified as pathogenic according to the ACMG criteria (PP3, PP2, PM2, PM1, PM5 and PS2).

Segregation analysis performed on the parents confirmed the *de novo* nature of the variant.

Genetic analysis also revealed the presence of a germline heterozygous variant c.507delT (p.Phe169LeufsTer2, rs587780183) in the *CHEK2* gene (NM_007194.3). The analysis of this gene was recently included in the panel of genes studied in patients with MB at our center, as an association between *CHEK2* and MB is reported in the literature, although not well established (Shah and Walter, 2018). The variant was inherited from the patient’s father and has previously been reported as likely pathogenetic, associated with a hereditary cancer-predisposing syndrome (Manoukian et al., 2011).

NGS was also performed on genomic DNA extracted from the tumor sample. Sequencing analysis revealed a somatic variant (allele burden 40%) in *PTEN*, c.388C > G (p.Arg130Gly) in addition to the germline change p.Tyr27Asn (allele burden 48%). This variant has been reported in the literature in individuals with clinical features characteristic of a *PTEN*-related disorder and identified as somatic variant in multiple malignancies (Fusco et al., 2020). The p.Arg130Gly variant affects *PTEN* function abolishing the phosphatase activities (Han et al., 2000). The variant in *CHEK2* was also found in the tumor sample with an allele burden of 46%.

Post-surgical chemotherapy was performed according to the Italian Association of Pediatric Hematology and Oncology MB infant reccomandations. It consisted in three courses of induction chemotherapy (methotrexate 8 g/m2 plus vincristine 1.5 mg/m2 week 0; etoposide 2.4 g/m2 week 1; cyclophosphamide 4 g/m2 plus vincristine 1.5 mg/m2 week 4) and two courses of high-dose thiotepa (300 mg/m2 for 3 days, week 7 and 12) followed by autologous hematopoietic stem cell transplantation (Massimino et al., 2013). Four years after diagnosis, the child is currently in remission from MB.

**Figure 2:**
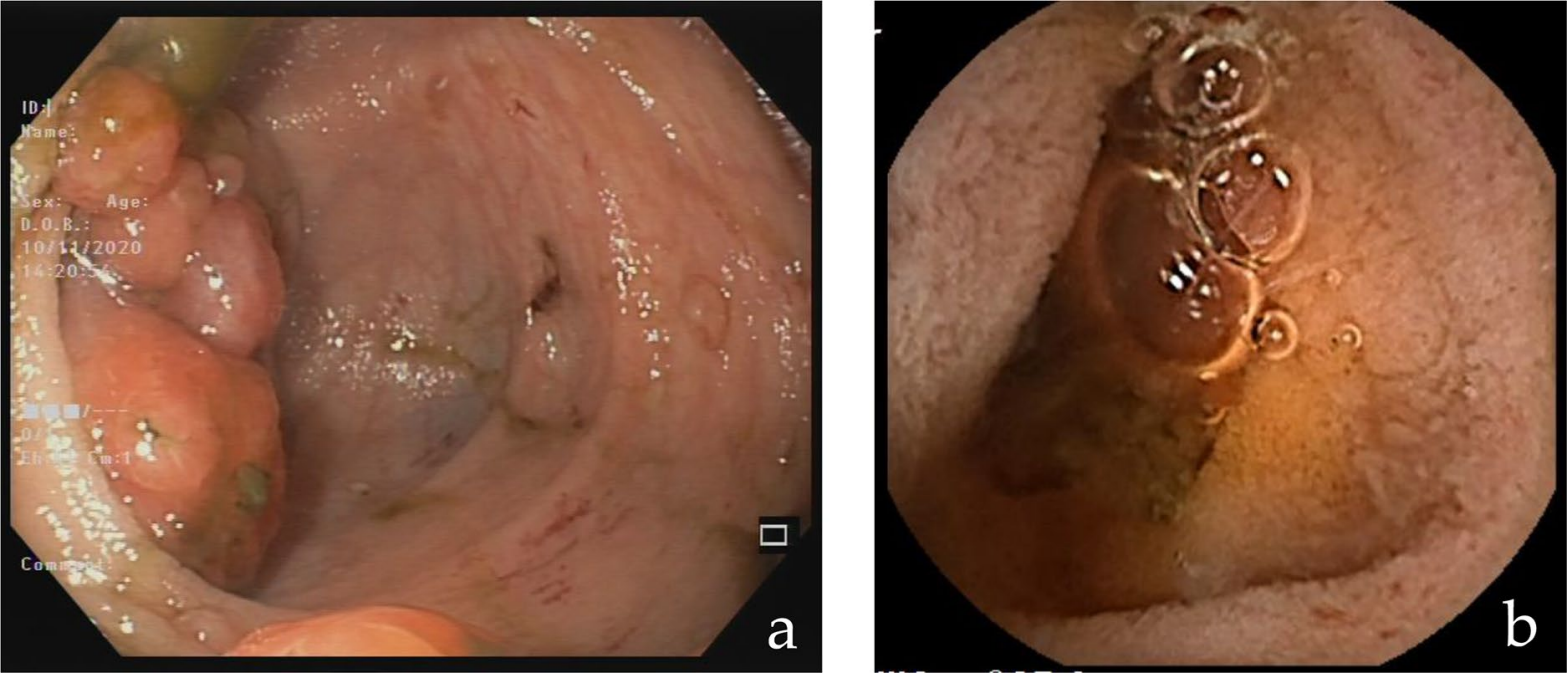
Endoscopic picture **(A)**: colon macro-polyp. Capsular picture **(B)**: middle ileum micro-polyp.

At 3 years of age, the patient presented with blood and mucus in stools, inappetence, recurrent abdominal pain and weight loss. For these reasons a colonoscopy was performed, and colic polyposis was found (>50 sessile lesions, others pedunculated). Some skin lesions compatible with PHTS (Tan et al., 2011) were also found: punctate keratosis of the palm-plantar region, hyperkeratotic papular lesions on the back of the hands and feet (trichilemmomas), numerous papular lesions on the back of the feet, periungual and axillary (acrochordon), papillomatous lesions of the oral cavity, and a melanocytic lesions in the abdominal region (compound melanocytic nevus). The child underwent the removal of 30 polyps (diameter 2 cm), during two endoscopic sessions; all polyps were hamartomatous (Figure 2).

At 4 years of age, an additional brain lesion (a right frontal with dural implant amartoma) was diagnosed and removed. The following year (at 5 years of age) severe hypoglycaemia was found and the patient needed to positionate a sensor and initially started feeding with the nasogastric tube. Severe hypoglycemia, although not always present in PTHS, is described in the literature as part of the clinical picture [always linked to *PTEN* regulation of the PI3K-AKT/mTOR pathway (Maines et al., 2021)].

## 4. Discussion

We present the first known case of a child carrying a germline and somatic pathogenic variant of *PTEN* associated with a germline and somatic variant of *CHEK2*, with a phenotype characterized by macrocephaly, DD, skin lesions, and very early onset of MB SHH and intestinal polyps.

There are only a few other cases with this type of MB-associated mutation that have been described: two pediatric cases, both with MB SHH (Table 1), and two cases of young adults (19 and 23 years) with MB SHH (Gröbner et al., 2018). It should be noted that the latter two patients are part of the series of Gröbner et al. (2018), which also includes a 4 year-old MB G3 patient with a *PTEN* low allele frequencies, in whom genetic analysis was performed only at the somatic level. Several studies have shown that *PTEN* variants are associated in 5% of cases with the development of CNS tumors (Liaw et al., 1997; Lynch et al., 1997; Staal et al., 2002; Sturm et al., 2014; Yakubov et al., 2016; Gröbner et al., 2018; Waszak et al., 2018; Kim et al., 2020), in particular glioblastoma, meningioma, dysplastic gangliocytoma, pineal tumor, and oligodendroglioma. The data currently available in the literature, although scarce, would suggest a possible association with MB as well. In contrast, it is not surprising that all patients with PHTS-associated MB belonged to the SHH subgroup, as more than 40% of pediatric SHH MBs have damaging germline mutations (Garcia-Lopez et al., 2021). The peculiarity of our case lies in the early onset of the brain tumor [presented before the first peak incidence of 3–4 years according to Millard and De Braganca (2016)], and the very early onset of the gastrointestinal manifestations, which usually occur in adulthood. Even cases of juvenile intestinal polyps in patients younger than 12 years with PHTS are rarely reported (Table 2). In fact, to our knowledge, this is the sixth described case of intestinal polyps in PHTS before the age of 12 years, and our patient is the youngest case reported so far. Due to the rarity of pediatric age intestinal manifestations, considering that even the National Comprehensive Cancer Network (NCCN) guidelines recommend “starting at 35 years old, unless symptomatic or close relative with colon cancer under age 40 years” a follow up with colonoscopy was not initially set (Daly et al., 2020).

**Table 1:** Known pediatric cases of MB with germline variants of *PTEN*.

Age at diagnosis	Sex	Medulloblastoma subtype	Gene variant	Other PHTS features
^a^15 months	M	SHH	*p*.Tyr27Asn	Macrocephaly, DD, bowel polyps, papillomas, trichilemmomas, acrochordon
^b^12 months	F	SHH	*p*.(Thr286ProfsTer5)	Unknown
^c^14 months	F	SHH	*p*.(Glu7Argfs*4)	Macrocephaly

^a^Our case; ^b^Waszak et al. (2018); ^c^Tolonen et al. (2020); DD, developmental delay; F, female; M, male; PHTS, *PTEN* hamartoma tumor syndrome.

**Table 2:** Known cases of affected by PHTS with bowel polyps’ onset before age of 12 years.

Age at diagnosis	Sex	PNET variant	Other PHTS features
^a^3 years	M	*p*.Tyr27Asn	Macrocephaly, DD, papillomas, trichilemmomas, acrochordon
^b^9 years	M	*p*.Phe337Ser	Macrocephaly, lipoma, tongue lesions, penile macules
^b^11 years	M	c.634 + 5G > A	Macrocephaly, lipoma, penile macules
^c^6 years	M	del(10)(q23.2q23.33)	Macrocephaly, DD, penile macules
^d^12 years	M	unknown	Lips polypoid excrescences, tonsillar papillomatosis
^e^4 years	M	del(10)(q23)	Macrocephaly, DD

^a^Our case; ^b^Lachlan et al. (2007); ^c^Tsuchiya et al. (1998); ^d^Gorensek et al. (1984); ^e^Hiljadnikova Bajro et al. (2013); DD, developmental delay; M, male; PHTS, *PTEN* hamartoma tumor syndrome.

Nearly 90% of patients with PHTS develop clinical manifestations before 20 years of age, although they may not be diagnosed until 30 years (Kim et al., 2020). There is an increased risk of developing breast or endometrial cancer for women, and thyroid cancer for both men and women. Colorectal cancer is also seen in 9–13% of cases, while polyps are found in 40–60% of cases (Heald et al., 2010). Other cancers were detected in the kidney and skin (Tan et al., 2011; Ngeow and Eng, 2015).

The *PTEN* protein acts as a potent suppressor of oncogenesis by inhibiting the PI3K-AKT/mTOR pathway and regulating cell proliferation and survival (Mester and Eng, 2013). Reinforcing the hypothesis that inhibition of this trail plays a crucial role in tumor pathogenesis. A study recently reported a reduction in hamartomas in patients with PHTS after rapamycin treatment, suggesting that patients with disorders in the *PTEN* hamartoma tumor syndrome spectrum might respond to therapies designed to inhibit the PI3-K/mTOR pathway (Marsh D. J. et al., 2008).

The SHH and PI3K pathways converge to promote the proliferation of granule cell progenitors in the outer granular layer of the cerebellum *in vitro* (Kenney et al., 2004). It has been observed in a mouse model that inactivation of the *PTEN* gene creates an abnormal perivascular proliferative niche in the cerebellum, persistent in adult animals, characterized by undifferentiated cells but without the tendency for malignancy, and in the absence of *TP53* or *PTCH1* codeletion (Zhu et al., 2017). Alterations in *PTEN* could therefore create a predisposing substrate for the development of MB, especially the SHH subgroup. A genomic analysis of medulloblastoma tumors showed that of 13 SHH subgroup patients, 2 had loss-of-function somatic mutations in *PTEN* (Robinson et al., 2012). Of 66 patients profiled from the other subgroups, none had loss of *PTEN*. Another study found a number of *PTEN* mutations in medulloblastoma tumors, one of which co-occurred with a homozygous PTCH mutation (Parsons et al., 2011). In addition, epigenetic inactivation of *PTEN* has been reported to occur at a high frequency in medulloblastoma samples (Hartmann et al., 2006). In our patient, sequencing analysis on tumor revealed the well-characterized loss of function somatic variant of *PTEN* p.Arg130Gly, that together with the germline missense change p.Tyr27Asn likely determines the complete loss of phosphatase activities of the protein, providing a strong evidence that the MB in our patient is associated with PHTS. On the contrary, we did not observe a loss of heterozygosity or the presence of a second deleterious somatic variant in *CHEK2*, suggesting this gene could have a marginal role in the tumorigenesis in our patient.

The increased susceptibility to develop hamartomatous polyps in the gastrointestinal tract is also related to uncontrolled cell growth in patients with *PTEN* mutation, especially subjects with heterozygous *PTEN* deletions developing intestinal epithelial dysplasia with subsequent invasion of the lamina propria, as described in adenoma-carcinoma progression (Marsh V. et al., 2008). The occurrence of bowel polyps has been described especially in patients with overlapping phenotypes between JPS and PTHS. *BMPR1A*, the gene associated with JPS, shares the same chromosomal region as *PTEN* (10q23.2): if large deletions encompass these genes the phenotypic expression can include features of both PHTS and JPS, most typically with juvenile polyposis of infancy (JPI), an aggressive subtype of JPS characterized by severe gastrointestinal symptoms, including diarrhea, intestinal bleeding, rectal prolapse, protein-losing enteropathy with a high risk of intussusception and consequently high infant mortality (Jelsig et al., 2014). The severity of this condition was hypothesized to be due to the loss of these two tumor suppressors, which function in two different but cooperative pathways (Delnatte et al., 2006; Salviati et al., 2006; Menko et al., 2008; Hiljadnikova Bajro et al., 2013). Our case did not present overlapping mutations between these genes, but rather a variant in the *CHEK2* gene.

The frameshift variant in the *CHEK2* gene, related to the *TP53* pathway, has been previously described in an Italian family with hereditary breast/ovarian cancer (HBOC) and is considered to be likely pathogenetic for cancer predisposition syndromes. However, the role of this variant is not yet fully understood, and it might be speculated that it elicits its effect in a context of polygenic inheritance, contributing to cancer risk in association with other susceptibility alleles and increasing the oncological recurrence risk in the family (Manoukian et al., 2011; Teodorczyk et al., 2013).

## 5. Conclusion

Although the association is rare, the panel of genes to be tested in the presence of an MB SHH could be extended to *PTEN*. The role of *CHEK2*, instead, remains uncertain at this time. The discovery of a *PTEN* germline mutation, even if in childhood, should induce the clinician to promptly provide genetic counseling in order to assess and monitor the occurrence of other PHTS clinical features and set up careful surveillance.

## Author contributions

AMC, AM, EA, and LB: conceptualization. AMC and SR: investigation and writing—original draft preparation. AMC, SR, LB, EA, MM, and AntC: data curation. AM, LB, EA, MM, FT, GSC, and AT: writing—review and editing. All authors have read and agreed to the published version of the manuscript.

## Conflict of interest

The authors declare that the research was conducted in the absence of any commercial or financial relationships that could be construed as a potential conflict of interest.

## Publisher’s note

All claims expressed in this article are solely those of the authors and do not necessarily represent those of their affiliated organizations, or those of the publisher, the editors and the reviewers. Any product that may be evaluated in this article, or claim that may be made by its manufacturer, is not guaranteed or endorsed by the publisher.
